# Apoptosis-inducing factor downregulation increased neuronal progenitor, but not stem cell, survival in the neonatal hippocampus after cerebral hypoxia-ischemia

**DOI:** 10.1186/1750-1326-7-17

**Published:** 2012-04-25

**Authors:** Yanyan Sun, Yu Zhang, Xiaoyang Wang, Klas Blomgren, Changlian Zhu

**Affiliations:** 1 Center for Brain Repair and Rehabilitation, Institute of Neuroscience and Physiology, Sahlgrenska Academy, University of Gothenburg, Gothenburg, Sweden; 2 Department of Pediatrics, The Third Affiliated Hospital of Zhengzhou University, Zhengzhou, China; 3 Department of Pediatrics, Zhengzhou Children’s Hospital, Zhengzhou, China; 4 Perinatal Center, Institute of Neuroscience and Physiology, Sahlgrenska Academy, University of Gothenburg, Gothenburg, Sweden; 5 Department of Pediatrics, The Queen Silvia Children’s Hospital, University of Gothenburg, Gothenburg, Sweden; 6 Department of Women’s and Children’s Health, Karolinska Institutet, Karolinska University Hospital, Stockholm, Sweden

**Keywords:** Apoptosis-inducing factor, Apoptosis, Asphyxia, Hippocampus, Neurogenesis, Cell proliferation

## Abstract

**Background:**

A considerable proportion of all newly generated cells in the hippocampus will die before becoming fully differentiated, both under normal and pathological circumstances. The caspase-independent apoptosis-inducing factor (AIF) has not been investigated previously in this context.

**Results:**

Postnatal day 8 (P8) harlequin (Hq) mutant mice, expressing lower levels of AIF, and wild type littermates were injected with BrdU once daily for two days to label newborn cells. On P10 mice were subjected to hypoxia-ischemia (HI) and their brains were analyzed 4 h, 24 h or 4 weeks later. Overall tissue loss was 63.5% lower in Hq mice 4 weeks after HI. Short-term survival (4 h and 24 h) of labeled cells in the subgranular zone was neither affected by AIF downregulation, nor by HI. Long-term (4 weeks) survival of undifferentiated, BLBP-positive stem cells was reduced by half after HI, but this was not changed by AIF downregulation. Neurogenesis, however, as judged by BrdU/NeuN double labeling, was reduced by half after HI in wild type mice but preserved in Hq mice, indicating that primarily neural progenitors and neurons were protected. A wave of cell death started early after HI in the innermost layers of the granule cell layer (GCL) and moved outward, such that 24 h after HI dying cells could be detected in the entire GCL.

**Conclusions:**

These findings demonstrate that AIF downregulation provides not only long-term overall neuroprotection after HI, but also protects neural progenitor cells, thereby rescuing hippocampal neurogenesis.

## Background

Perinatal asphyxia-induced brain injury is one of the most common causes of mortality and long term neurological impairment (cerebral palsy, mental retardation, visual as well as hearing impairment, learning disability and epilepsy) in term and preterm neonates [1]. A significant breakthrough was that post-insult hypothermia within 6 hours reduced severe disability, including cerebral palsy [2]. Other milestones in the field were the findings that low dose rhEPO treatment [3] or delayed hypothermia up to 10 hours [4] reduce the risk of disability in infants with moderate term hypoxic-ischemic encephalopathy. Current therapeutic options for preterm hypoxic-ischemic brain injury are limited and predominantly supportive, to maintain physiological parameters.

Hypoxia-ischemia (HI) causes opposite reactions of injury and repair. On the one hand, HI leads to neuronal, including neural stem/progenitor cell (NSPC), death and brain injury by excessive production of free radicals, excitatory amino acids, inflammation as well as mitochondrial dysfunction [1]. The patterns of neuronal cell death after HI involve necrosis, apoptosis and autophagy, based on biochemical and morphological criteria, and accumulating data show that mixed morphological phenotypes often are observed [5-9]. The immature brain retains its apoptotic machinery to a larger extent than the adult brain, at least as judged by caspase-3 activation [10-13]. There are two distinct pathways leading to nuclear apoptosis, caspase-dependent and caspase-independent. Caspases are a class of cysteine proteases that mediate apoptotic death in a variety of cellular systems. Caspases are activated after HI, particularly in the immature brain [12]. Consequently, caspase inhibition affords neuroprotection in the immature brain [14,15]. Recent data demonstrate that when caspase activation is inhibited at or downstream of the apoptosome, neurons can undergo a delayed, caspase-independent death [16]. One of the key components of the caspase-independent cell death pathway is apoptosis-inducing factor (AIF) which, when released from mitochondria, translocates to the nucleus and induces large-scale DNA fragmentation and cell death [17]. Inhibition or depletion of AIF can provide neuroprotection *in vitro* and *in vivo*[14,18,19]. HI also activates protective and restorative mechanisms. For example, HI up-regulates erythropoietin (EPO) and other growth/trophic factors and induces NSPC proliferation and differentiation [20]. Proliferating cells migrate to the injury [21-23], even very long after the insult [24,25], inspiring hope for tissue repair and plasticity. However, a related problem is the poor survival of the newly generated NSPCs after ischemic insults [26]. More than 90% of newborn cells die within one month [27]. Therefore, exploring death mechanisms of endogenous NSPCs will provide important information for prevention/inhibition of NSPC death and hopefully promote brain repair. The purpose of this study was to evaluate the effects of AIF downregulation on neuronal and NSPC death after HI in the immature brain as well as long-term effects on brain injury.

## Results

### AIF downregulation reduced HI brain injury

**Figure 1:**
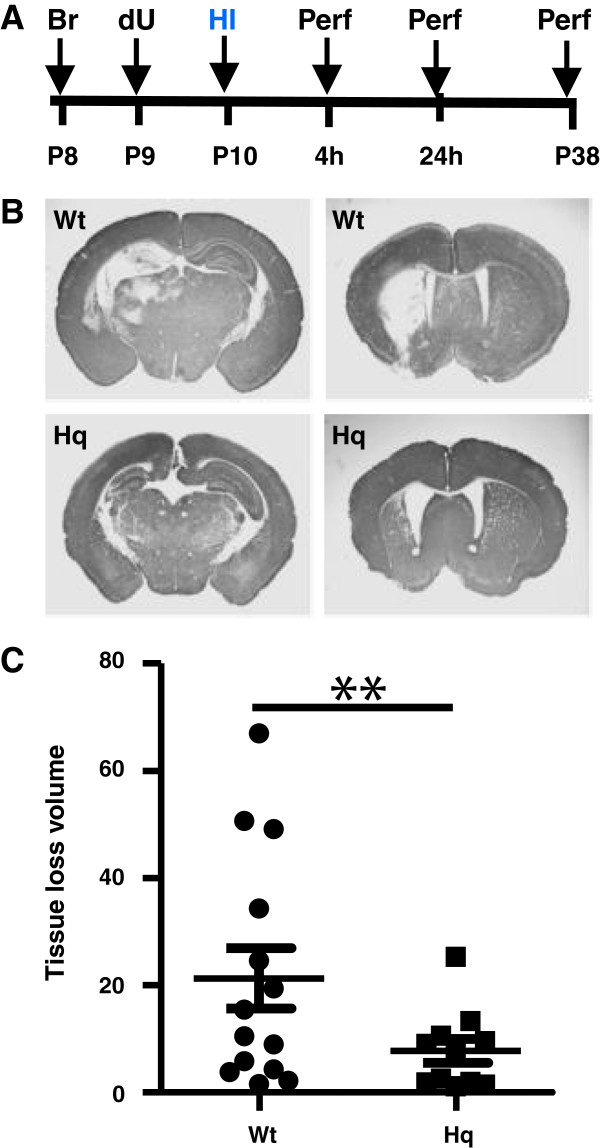
**AIF down regulation reduced HI brain injury. A.** The study design. **B**. Representative MAP2 stainings from the levels of dorsal hippocampus (left panels) and striatum (right panels) 4 weeks post-HI of wild type (Wt) (upper panels) and Harlequin mutant (Hq) mice (lower panels). **C**. The total tissue loss volume 4 weeks after HI was reduced by 63.5% in Hq mice (n=11) as compared with Wt mice (n=14). **P<0.01.

Brain injury was evaluated on P38, 4 weeks after HI (Figure 1A). The injury encompasses the cortex, hippocampus, striatum and thalamus (Figure 1B). The overall tissue loss, including infarction and subsequent atrophy, was 21.3±5.6 mm^3^ (n=14) in Wt mice and 7.8±2.2 mm^3^ (n=11) in Hq mutant mice, which corresponds to a 63.5% reduction (P=0.002) (Figure 1C).

### AIF downregulation had no effect on short-term proliferation and survival

**Figure 2:**
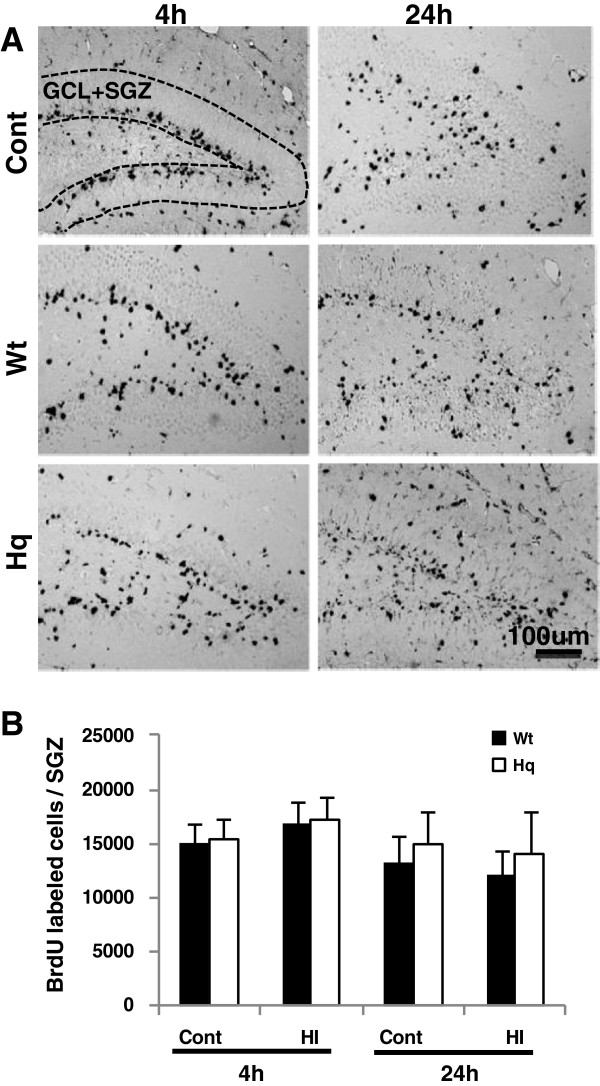
**Cell proliferation in the DG after HI. A**. Representative microphotographs of BrdU stainings in the DG in naïve controls (top panels), at 4 h (left panels) and 24 h (right panels) after HI. **B**. The bar graph shows quantification of the total number of BrdU-positive cells in the SGZ in controls (Cont) and ipsilateral hemisphere after HI in Wt and Hq mice 4 h (n=5 or 8 for Wt and Hq controls, n=6 or 7 for Wt and Hq HI) and 24 h (n =6 or 8 Wt and Hq controls, n=7 or 8 for Wt and Hq HI).

Cell proliferation in the subgranular zone (SGZ) was assessed 4 h and 24 h after HI by BrdU labeling 1 and 2 days before HI (Figure 1A). This means that the labeled cells were born 1 or 2 days before HI and survived at least until 4 h or 24 h after HI. Neither the HI insult nor the Hq mutation had any obvious short-term effect on the number of BrdU-labeled cells in the SGZ (Figure 2A, 2B).

### AIF downregulation increased neuronal progenitor, but not stem cell, survival

**Figure 3:**
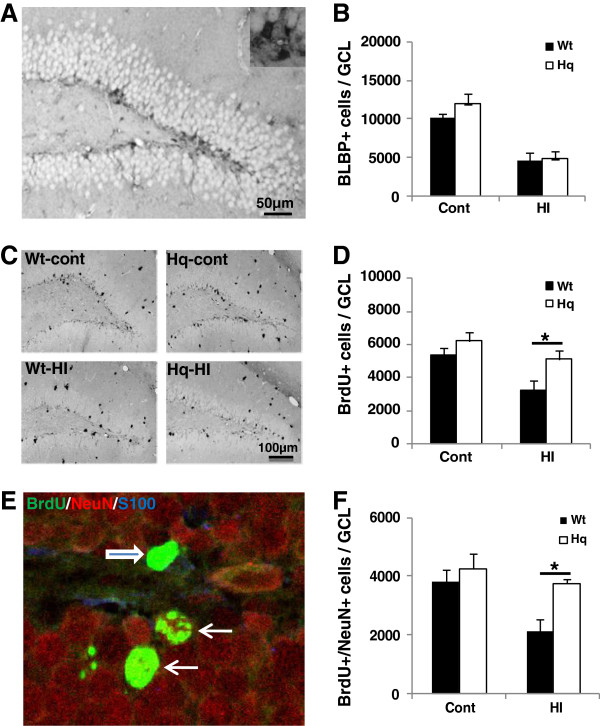
**Survival and differentiation of proliferated cells in the DG 4 weeks after HI. A**. Representative microphotograph of BLBP staining in the DG 4 weeks after HI. **B**. The bar graph shows quantification of the total number of BLBP-positive cells in the SGZ. **C**. Representative microphotographs after BrdU staining in the DG at 4 weeks after HI. **D**. Quantification of BrdU-positive cells in the DG (GCL+SGZ) in the control (Cont) and ipsilateral hemisphere after HI in Wt and Hq mice. **E**. Representative microphotograph showing triple labeling of BrdU/NeuN/S100 in the DG. **F**. Quantification of the number of BrdU/NeuN double-positive cells (neurons) in DG (GCL) in the control (Cont) and ipsilateral hemisphere after HI in Wt and Hq mice. * P<0.05.

Cells in the SGZ expressing brain lipid binding protein (BLBP) are undifferentiated, radial glia-like neural precursors [28]. The number of BLBP-labeled cells in the SGZ was not different between Wt and Hq mice, neither in control brains, nor after hypoxia-ischemia (Figure 3A3B). The total number of surviving BrdU-labeled cells per GCL in the control was 5359±456 in Wt (n=5) and 6210±541 in Hq (n=6) brains after 4 weeks (n.s.). However, the number of BrdU-labeled cells surviving 4 weeks after HI was higher in Hq than Wt brains (p=0.030). In Wt mice, the number decreased 40.1%, to 3209±605 (p=0.013), whereas in Hq mice the decrease was only 17.6%, to 5115±532 (n.s.) (Figure 3C3D). The phenotyping of newly generated cells surviving 4 weeks showed that about 70% developed into neurons, as judged by BrdU/NeuN double labeling (Figure 3E3F), and this ratio was the same in Wt and Hq animals, both in non-ischemic control and HI brains. Importantly, while the number of labeled cells surviving 4 weeks after HI and differentiating into neurons decreased approximately 45% in Wt mice, it was not significantly decreased in Hq mice (Figure 3F). The number of newly generated astrocytes, as indicated by BrdU/S100 double labeling was not different, neither between genotypes, nor between control and HI mice (data not shown).

### AIF downregulation reduced cell death in the dentate gyrus

**Figure 4:**
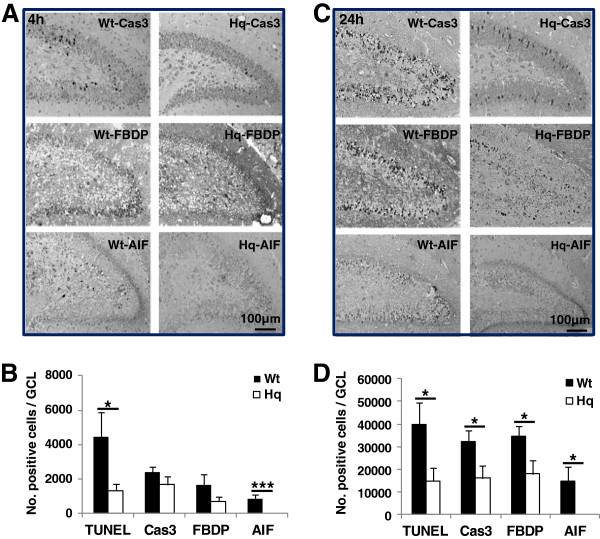
**Cell death in the DG after HI. A.** Representative stainings of active caspase-3, FBDP and AIF in the DG of Wt (left panel) and Hq (right panel) mice 4 h after HI. **B**. The bar graph shows the quantification of positive cells for each cell death-related marker in the GCL at 4 h after HI. **C**. Representative stainings of active caspase-3, FBDP and AIF in the DG of Wt (left panel) and Hq (right panel) mice 24 h after HI. **D**. The bar graph shows the quantification of positive cells for each cell death-related marker in the GCL at 24 h after HI * P<0.05, *** P<0.001.

Cell death in the hippocampus was investigated by detecting DNA strand breaks (TUNEL), necrosis (FBDP), as well as caspase-dependent (active caspase-3) and -independent (nuclear AIF) apoptosis [12]. The number of positive cells was counted in the GCL+SGZ 4 h and 24 h after HI (Figure 4). Interestingly, early after HI (4 h) dying cells, as judged by TUNEL, active caspase-3 and nuclear AIF, were mainly detected in the inner layers of the GCL, whereas later (24 h) after the insult dying cells could be detected throughout the GCL (Figure 4A). Necrotic (FBDP-positive) cells, though, were mainly detected in the outer layers of the GCL, in differentiated neurons rather than immature NSPCs, both 4 h and 24 h after HI (Figure 4). The total number of dying cells was higher in Wt mice, particularly 24 h after HI (Figure 4D). There were no AIF-positive cells, displaying nuclear AIF, in Hq mice (Figure 4).

### AIF downregulation reduced newborn cell death

**Figure 5:**
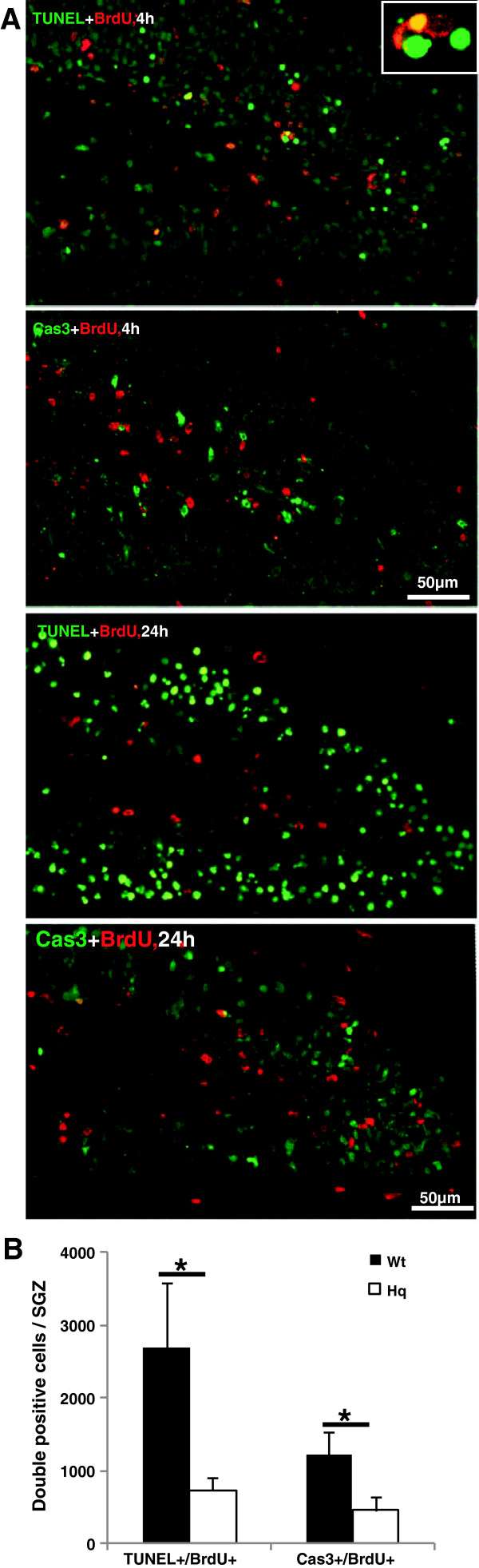
**Double labeling of BrdU and cell death markers after HI. A**. Representative double labelings of BrdU with the cell death-related markers TUNEL and active caspase-3 in the DG 4 h and 24 h after HI. **B**. The number of dying newborn cells in the SGZ, as indicated by double labeling 4 h after HI, was lower in the Hq mice. * P<0.05.

Death of newly generated cells was detected by combining BrdU staining with the cell death markers TUNEL or active caspase-3 4 h and 24 h after HI (Figure 5A). The total number of TUNEL/BrdU double-positive cells in the SGZ was 2691±894 in the Wt mice and 731±186 (n=6) in the Hq mice (n=7) (p=0.041) 4 h after HI. The total number of active caspase-3/BrdU double-positive cells was also lower in Hq mice (453±197) compared with Wt mice (1229±304) (p=0.0498) (Figure 5B). In the Hq mice, fewer cells were dying, but the overall numbers of BrdU-labeled cells in the SGZ 4 h and 24 h after HI were not different between Hq and wild type mice, as shown above (Figure 2B). The double-positive cells were fewer in the SGZ 4 h than 24 h after HI and no significant differences between Wt and Hq mice were observed (data not shown).

### AIF downregulation reduced pathological microglia activation

**Figure 6:**
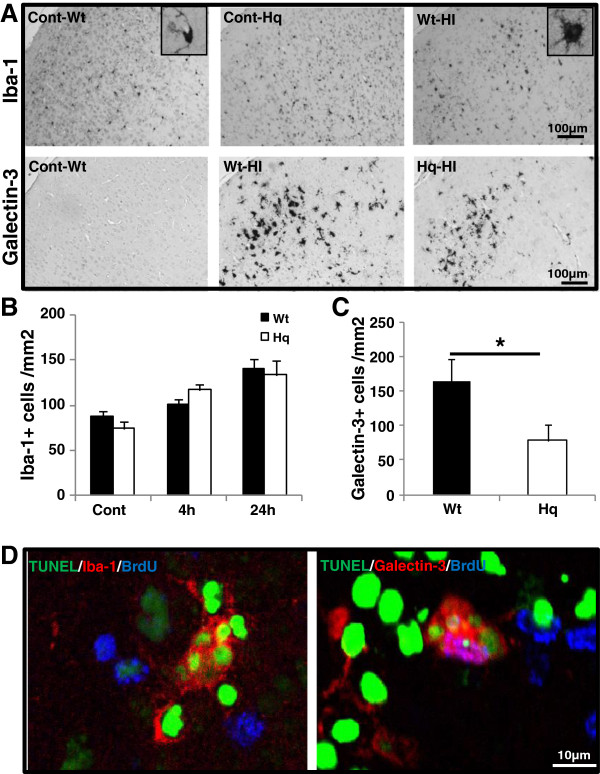
**Microglia activation after HI. A**. Representative Iba-1 (upper panel) and galectin-3 (lower panel) immunostainings in the cortex of both Wt and Hq control brains and 24 h after HI. **B**. Quantification of Iba-1-positive cells in the cortex did not show any difference between Wt and Hq mice. **C**. Quantification of galectin-3-positive cells in the cortex 24 h after HI showed fewer pathologically activated microglia in the Hq mice. **D**. Triple labeling with TUNEL, BrdU and Iba-1 or galectin-3 24 h after HI showing Iba-1- or galectin-3-positive cells engulfing dead cells (TUNEL-positive chromatin fragments).

The total numbers of microglia in the immature Wt or Hq brains were not different, according to Iba-1 immunostaining and quantification (Figure 6A6B). Galectin-3, a marker of pathological microglia activation [29], was not detected in the normal control brains, it was detected only in injured areas, as expected (Figure 6A). The number of galectin-3-positive cells was lower in the Hq mice than in Wt mice after HI (Figure 6C). Some of the activated microglia were observed engulfing dying cells, as judged by TUNEL-positive chromatin fragments inside these cells (Figure 6D).

## Discussion

Apoptosis is important during normal brain development [30,31]. Our previous studies have shown that apoptotic cell death after HI in the immature brain involves caspase-dependent and –independent pathways [14]. Caspases are activated after HI, particularly in the immature brain [12] and caspase inhibition affords neuroprotection in the immature brain [14,15,32]. A causal role of AIF for neuronal cell death and brain injury following HI in the immature brain has been identified by using Hq mutant mice, where brain injury was reduced by 52.6% at 3 days after HI [14]. In this study we demonstrate long-term neuroprotection in AIF-deficient mice after HI, where brain injury was reduced by as much as 63.5% 4 weeks after HI. This extends our earlier findings and further confirms that AIF plays a major role in the development of brain injury after HI. After HI, a large proportion of the dying neurons in the immature brain are immunopositive for both active caspase-3 and nuclear AIF, but there are also populations of neurons displaying only active caspase-3 or only nuclear AIF [18]. We found that Hq mice displayed approximately 50% smaller infarct volumes than wild type mice. The caspase inhibitor Q-VD-OPh also produced approximately 50% smaller infarct volumes, and combining the two, by treating Hq mice with Q-VD-OPh, produced an additional 50% infarct volume reduction [14]. Also in a model of traumatic brain injury in adult mice, increased protection was found when combining functional AIF reduction (as in *cyclophilin A*^*−/−*^ mice) with caspase inhibition (boc-Asp-FMK) [33]. Hence, caspase-dependent and caspase-independent (AIF-dependent) pathways appear to act, at least partly, in parallel.

Opposite its role in mediating apoptosis once it is released from mitochondria, AIF, as a flavoprotein, is essential for the maintenance of a fully functional complex I [34]. In healthy cells, the physiological role of AIF in sustaining complex I-driven oxidative phosphorylation appears related to the local redox activity of AIF and is independent of its proapoptotic properties [35]. Efforts to study the mitochondrial function of AIF have focused on the putative ability of AIF to regulate reactive oxygen species [36]. Genetic mutant Hq mice with up to 80% reduction of mitochondrial AIF, display reduced levels of complex I and impaired assembly of complex I subunits [37]. These mice exhibit mitochondrial respiratory chain diseases, such as cerebellar neurodegeneration with ataxia and progressive retinal degeneration. A recent study showed that the mitochondrial complex I contributes to oxidative injury during early reperfusion after HI in the neonatal mouse brain and that inhibition of complex I decreased the extent of HI injury [38]. The Hq mutation, displaying reduced levels of AIF, but also reduced levels of for example catalase and complex I, renders the brain tissue more susceptible to oxidative stress [14,37]. We did not assess the levels of oxidative stress in the SGZ in this study, but it would be interesting to evaluate the effects of an antioxidant agent, to see if the effects in the SGZ would be different from the effects in mature neurons.

Interestingly, we observed a wave of apoptotic cell death starting at the inner layers of the GCL and moving outwards, and at the same time increasing about 10-fold in numbers of dying cells from 4 h to 24 h after HI (Figure 4). Necrotic cell death, as judged by massive calcium influx leading to calpain-specific cleavage of fodrin to yield the 150 kDa breakdown product (FBDP), was only observed in the outer layers of the GCL, indicating that necrosis occurred only in fully differentiated neurons, not in stem and progenitor cells. Only a fraction of the BrdU-labeled cells in the inner layers of the GCL died (underwent apoptosis) after HI. This means that out of all the BrdU-labeled cells in the SGZ, born 1 or 2 days earlier and surviving until 4 h after HI, approximately 16% were TUNEL-positive and 7% were active caspase-3-positive (compare Figures 2B and 5B). In the Hq mice, approximately 70% fewer cells were dying (were TUNEL-positive) in the SGZ after HI, but the overall numbers of BrdU-labeled cells (dying and not dying) in the SGZ 4 h and 24 h after HI were not different between Hq and wild type mice (Figure 2B). The double-positive cells were fewer in the SGZ 24 h than 4 h after HI and no significant differences between Wt and Hq mice were observed (data not shown). As noted above, by this time point (24 h) the wave of cell death had spread outward and, consequently, fewer dying, newly generated cells could be detected in the inner layers (SGZ), even though the total number of dying cells was approximately 10 times higher in the entire GCL at this later time point. It is not clear why the apparently lower rate of dying cells in the SGZ in Hq mice did not lead to a difference in undifferentiated, BLBP-positive cells 4 weeks later (Figure 2A2B). Presumably, the AIF downregulation only protected neuronally committed progenitor cells and neurons, not undifferentiated stem cells. Also, as mentioned above, it was only a fraction of the BrdU-labeled cells that were TUNEL- or active caspase-3-positive, and only a fraction of these that was protected by the AIF deficiency. Nevertheless, the loss of a number of undifferentiated as well as more or less differentiated cells after HI leads to impaired growth of the DG, resulting in a smaller DG volume 5 weeks after HI, as shown earlier [39]. Overall, we have shown that AIF plays an important role not only in the HI-induced death of mature neurons throughout the brain, but also in the HI-induced death of newborn cells and neuronal progenitors in the DG.

Neurogenesis can be induced by brain ischemia, indicating that regenerative mechanisms are activated by injurious stimuli. This could inspire hope for development of restorative therapies for neurological disorders and brain injuries. However, the majority of newborn cells appearing after HI do not survive beyond 3 weeks, as judged by BrdU-labeling [26,27]. Since immature cells are more prone to undergo apoptosis than fully differentiated neurons, particularly in the immature brain, we propose that the continuous and massive decrease in the number of BrdU-labeled cells induced by an injury may be due to apoptosis-mediated cell death, which in turn reduces the restorative capacity. A previous study showed that stem and progenitor cells in the subventricular zone die after HI at least partly through caspase-3 and calpain activation [6]. Under normal conditions in the adult hippocampus, the majority of newborn cells undergo death by apoptosis in the first 1 to 4 days of life, during the transition from amplifying neuroprogenitors to neuroblasts [40]. In the present study, we followed the fate of cells born 1 or 2 days before HI, i.e. when they were changing from transient amplifying cells to neuroblasts. Reducing apoptosis through overexpression of bcl-2 under the neuron-specific enolase (NSE) promoter was found to double the rate of neurogenesis in the dentate gyrus as demonstrated by quantification of doublecortin-positive progenitor cells and BrdU/NeuN double-labeling. The effect of Bcl-2 was limited to the late phase of progenitor maturation, presumably correlating with the onset of NSE expression, as proliferation and early-phase progenitor cells were not affected and the increased level of neurogenesis led to a significantly higher total number of granule cells in the dentate gyrus [41]. Pharmacological caspase inhibition could increase the number of surviving, seizure-induced newborn neurons [42]. The role of AIF in the death of newborn cells after HI in the immature brain has not been investigated before. In this study, we found evidence for AIF-induced cell death both in the early phase of transient amplifying cells/early neuroblasts as well as in the later phase of mature neuronal progenitors and young neurons.

## Conclusions

In summary, this study demonstrates that AIF plays a role in the wave of apoptotic cell death extending from the inner layers of the GCL outward and that downregulation of AIF could rescue hippocampal neurogenesis.

## Materials and methods

### Induction of HI brain injury

Postnatal day 10 (P10) wild type or Harlequin mutant (Hq) mouse pups were anesthetized with isoflurane (5% for induction, 1.5-3.0% for maintenance) in a mixture of nitrous oxide and oxygen (1:1); the duration of anesthesia was less than 5 min. The left common carotid artery was cut between double ligatures of prolene sutures (6.0). After the surgical procedures the wounds were infiltrated with lidocaine for analgesia. The pups were returned to their dams for 60 min and then placed in a chamber perfused with a humidified gas mixture (10% oxygen in nitrogen) for 45 min at 36°C to induce a mild brain injury [12]. Following hypoxic exposure, the pups were returned to their dams until sacrifice. Control pups were neither subjected to ligation nor hypoxia. All animal experimentation was approved by the Gothenburg Committee of the Swedish Animal Welfare Agency (145–2008).

### BrdU administration

The thymidine analog 5-bromo-2-deoxyuridine (BrdU) (Roche, Mannheim, Germany, 5 mg/mL dissolved in 0.9% saline) was prepared freshly prior to use and injected intraperitoneally (50 mg/kg) on P8 and P9, before HI (Figure 1A).

### Injury evaluation

Brain injury was evaluated by the volume of total hemispheric tissue loss, as judged by MAP2 immunostaining. The MAP2-positive and -negative areas in each section were measured using Micro Image (Olympus, Japan). The tissue volume was calculated from the MAP2-positive areas according to the Cavalieri principle using the following formula: V=ΣA×P×T, where V=total volume, ΣA=sum of area measurements, P=the inverse of the sampling fraction, and T=the section thickness. The total hemispheric tissue loss was calculated as the MAP2-positive volume in the contralateral hemisphere minus the MAP2-positive volume in the ipsilateral hemisphere.

### Immunohistochemistry

The animals were anesthetized and perfusion-fixed with 5% formaldehyde in 0.1 M PBS. The paraffin-embedded brains were serial cut in 5 μm coronal sections and mounted on glass slides. On the hippocampus level, every 50^th^ section was stained. Antigen retrieval was performed by heating the sections in 10 mM boiling sodium citrate buffer (pH 6.0) for 10 min. Nonspecific binding was blocked for 30 min with 4% goat or horse or donkey serum in PBS. Monoclonal rat anti-BrdU (1:100, 5 μg/ml; clone: BU1/75, Oxford Biotechnology Ltd. Oxfordshire, UK), monoclonal mouse anti-MAP-2 (1:1000, clone HM-2, Sigma, Saint Louis, Missouri, USA), rabbit anti-active caspase-3 (1:100, 10 μg/ml, BD Pharmingen, USA), rabbit anti-FBDP (1:50), goat anti-AIF (1:100, 2 μg/ml, sc-9416, Santa Cruz), rabbit anti-brain lipid binding protein (BLBP) (1:600, ABN14, Millipore, Temecula, CA, USA), rabbit anti-Iba-1 (1:1000, 0.5 μg/ml, Wako, Osaka, Japan), rat anti-galectin-3 (1:100, 5 μg/ml, eBioscience, San Diego, CA, USA) primary antibody was applied and incubated at 20°C for 60 min, followed by the appropriate biotinylated secondary antibodies for 60 min at 20°C. Visualization was performed using Vectastain ABC Elite kit (Vector Laboratories, Burlingame, CA, USA).

For TUNEL and BrdU double or triple labelings, after antigen retrieval, sections were incubated with 3% bovine serum albumin in 0.1 M Tris–HCl (pH 7.5) for 30 min followed by 50 μl of TUNEL reaction mixture on each sample for 60 min at 37°C in a moisture chamber. After washing, the sections were incubated with rat anti-BrdU (1:100, 5 μg/ml; clone: BU1/75, Oxford Biotechnology Ltd. Oxfordshire, UK) or mouse anti-BrdU and rabbit anti-Iba-1 or mouse anti-BrdU and rat anti-galectin-3 for 60 min at room temperature. After washing, the sections were incubated appropriate Alexa Fluor labeled donkey anti-rat IgG (H+L) or Alexa Fluor 555 donkey anti-rabbit and Alexa Fluor 647 donkey anti-mouse or Alexa Fluor 555 donkey anti-rat and Alexa Fluor 647 donkey anti-mouse at 20°C for 60 min. For active caspase-3 and BrdU double labeling, rabbit anti-active caspase-3(1:100, 10 μg/ml, BD Pharmingen, USA) and rat anti-BrdU (1:100, 1:100, 5 μg/ml) were incubated at 20°C for 60 min. After washing, the sections were incubated with Alexa Fluor 555 donkey anti-rat IgG (H+L), combined with Alexa 488 donkey anti-rabbit IgG (H+L).

The phenotype of BrdU-labeled cells was determined using antibodies against NeuN or S100β. The sections were incubated with rat anti-BrdU together with mouse anti-NeuN monoclonal antibody (1:200, 5 μg/ml; clone: MAB377, Chemicon, Temecula, CA, USA) and rabbit anti-S-100ß (1:1000; Swant, Bellinzona, Switzerland) in PBS at 20°C for 60 min. After washing, the sections were incubated with secondary antibodies, Alexa Fluor 488 donkey anti-rat IgG (H+L), combined with Alexa 555 donkey anti-mouse IgG (H+L) and Alexa 647 donkey anti-rabbit IgG (H+L) at 20°C for 60 min. All secondary antibodies were from Jackson ImmunoResearch Lab and were diluted 1:500. After washing, the sections were mounted using Vectashield mounting medium.

### Cell counting

The number of BrdU-labeled cells were counted in every 50^th^ section in the subgranular zone (SGZ) 4 h or 24 h after HI, or in the entire granule cell layer (GCL), including the SGZ, 4 weeks after HI, using unbiased stereological counting techniques (StereoInvestigator, MicroBrightField Inc., Magdeburg, Germany). For the phenotype, at least 50 BrdU-positive cells in the GCL were phenotyped using a confocal laser scanning microscope (Leica TCS SP, Heidelberg, Germany) and the ratio of Brdu/NeuN or BrdU/S100β double-labeled cells was calculated for each sample. The total number of neurons (BrdU/NeuN-positive) and astrocytes (BrdU/S100β-positive) in each sample was calculated based on the number of BrdU-positive cells and the ratio of double labeling. The BrdU and TUNEL or BrdU and active caspase-3 double-positive cells were counted in the SGZ by using confocal microscopy. The Iba-1- or galectin-3-positive cells were counted at 400x magnification in the border zone of the injured cortex within an area of 0.196 mm^2^. Three sections were counted from each brain with an interval of 250 μm. The average was used as n=1 when comparing different brains. All the counting was carried out by investigators blinded to group assignment.

### Statistical analysis

All data are expressed as mean±s.e.m. Student’s unpaired t-test was used to compare the numbers of cell death-related markers, BrdU-positive cell numbers, numbers of newborn neurons and astrocytes between the two groups. Significance was assumed when p<0.05.

## Abbreviations

AIF = Apoptosis inducing factor; BLBP = Brain lipid binding protein; BrdU = 5-bromo-2-deoxyuridine; DG = Dentate gyrus; FBDP = Fodrin brakdown product; GCL = Granule cell layer; HI = Hypoxia ischemia; Hq = Harlequin; MAP-2 = Microtubule associated protein-2; NSPC = Neural stem/progenitor cell; P = Postal day; PBS = Phosphate-buffer saline; SGZ = Subgranular zone; TUNEL = Terminal deoxynucleotidyl transferase mediated dUTP nick end-labeling; Wt = Wild type.

## Competing interests

The authors declare that they have no competing interests.

## Authors’ contribution

YS, YZ, XW and CZ performed experiments. CZ and KB conceived and designed the experiment and wrote the manuscript. YZ, YZ and CZ collected and assembled the data. All authors read and approved the final manuscript.
